# Soul Men and Women—what must science do to regain public trust?

**DOI:** 10.1038/s44319-024-00325-0

**Published:** 2024-11-20

**Authors:** Arthur Caplan

**Affiliations:** NYU Langone Medical Center, Division of Medical Ethics, New York, USA

**Keywords:** Economics, Law & Politics, History & Philosophy of Science

## Abstract

To meet the growing deluge of misinformation and distortion of scientific facts, science desperately needs a PR makeover.

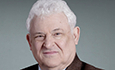

“No matter if it’s a severe winter storm, thunderstorm, tornado, hurricane, flash flood, or any other significant natural hazard, our job as broadcast meteorologists is to take the viewers by the hand and help them make informed decisions that could save their lives. Nobody in broadcast media has this responsibility on a daily basis. And there is no greater compliment than when somebody says ‘you saved my life.” Veteran American TV meteorologist

The USA’s two leading climate-focused channels saw a surge in viewership as multiple states braced for the impact of Hurricane Helene in late September 2024. Nearly two million people tuned in to watch The Weather Channel’s coverage during the 10 pm Eastern Time block on September 26. The public knows, trusts and turns to its most visible scientists in times of crisis—or maybe not.

A series of falsehoods and threats have swirled in the two weeks after Hurricane Helene tore through six states causing hundreds of deaths, followed by Milton crashing into Florida. Meteorologists have received a torrent of conspiracy theories that they were controlling the weather, abuse and even death threats, amid what they say is an unprecedented surge in misinformation as two major hurricanes hit the USA.

The extent of the misinformation, which has been stoked by Donald Trump and his followers, has been such that it has stymied the ability to help hurricane-hit communities, according to the head of the US Federal Emergency Management Agency.

Katie Nickolaou, a Michigan-based meteorologist, said that she and her colleagues have borne the brunt of much of these conspiracies. They have received messages claiming that meteorologists or the government are creating hurricanes—they are, in fact, not—and even that scientists should be killed and radar equipment be demolished. One post aimed at Nickolaou said: “Stop the breathing of those that made them and their affiliates” to which she responded that “Murdering meteorologists won’t stop hurricanes. I can’t believe I just had to type that.”

When your trusted local meteorologist becomes the target of abuse and death threats, when people call for the execution of the world’s most distinguished public-health scientists in the midst of a dangerous pandemic, it is fair to say that science has a big trust problem. Actually, the problem is worse than a loss of public trust among some. Science is now seen as just one more example of ideology—whether it is by conspiracy theorists believing that meteorologists fabricate hurricanes, by anti-vaxxers who think that vaccines kill people or by radical environmentalists who believe that all genetically modified organisms inevitably destroy the environment. Understanding the growing mistrust in and rejection of science may contain the keys to knowing how to fight it.

These versions of anti-science sentiments churning through modern culture are not new. For at least a century, some on the right and the left worried that science, initially due to evolutionary theory, and later, the development and use of the atomic bomb and the resulting ‘cold war’, represented a moral threat to civilization, due to its corrosive effects on humanity’s self-conception. Influential groups of American opinion leaders along with thinkers in other countries have long argued and still do that science is anchoring a faulty cultural understanding of human beings, finding and depicting them as soulless.

Our contemporary fights over science reflect the potent impact of this tradition which claims that science lacks values, takes a purposeless view of our existence and sees humans as automata deluded about free will but in reality completely controlled by genes, nutrition, conditioning, and culture. In addition, science is perceived as a tool of the elites—greedy business moguls, distant corporations, bureaucrats and the military—and that it is conducted by elites: inscrutable nerds who live and work, well paid, in ivory towers. Herein lie the clues about some of the actions science must take to recapture public trust and tamp down the ideological assaults contending that science robs humanity of its dignity and spirit.

What is required is more than just efforts to correct misinformation or to bring warranted evidence to bear on contentious issues. That work is hugely important, but it requires a parallel effort to reclaim science’s voice as a trusted source to succeed. No amount of facts will suffice to influence any controversy if the audience does not trust the messenger (Badur et al, 2020).

Science may find no purpose or meaning in what cosmology or evolutionary theory have to say about eternity and our place in it, the story of evolution may lack a special place for humans, but that hardly means that science is done by people who are soulless or amoral. In fact, as scientists all too rarely explain to anyone outside their communities, they are drawn to science by very moral desires and drives: to cure diseases that have ravaged their friends or families, fight starvation, to find more humane and less back-breaking ways to work, to protect the environment and preserve endangered species, to create the means to explore other planets and the deepest oceans, and so on. It is the results of successful science that produce meaning—and that ought to be widely communicated as exceedingly meaningful for practitioners of science. When a child with a life-threatening tumor comes home after therapy saved his life, or a woman who lost her arms uses a sophisticated prosthetic to hug her sister, these are moments full of emotion, spirit and, yes, soulfulness for the scientists who developed the therapy or the prosthetics.

Whether it amounts to anything in the very long run, preserving this planet and its occupants safely for the next hundred years ought to provide enough soul and meaning to anyone seeking value from science. But science must empower and boost voices who can speak with humility and emotion about what they do and the results of their work.

Similarly, the view that science is done by unrelatable people in obscure places is correctable if only scientists would take the debunking of this distorted view seriously. Young scientists are absolutely relatable. If they are told they must make the public understand what they do part of their scientific duty, they would eliminate the gap between a mystified public and the all-too-human and humane practitioners of science. One talk every year given at a high school, primary school, Rotary, Sunday school, or retirement home would be a great start. And bringing the community to science facilities and research parks, explaining who works there, what they do and their impact including the economic impact on the local community would not hurt either. Science needs a PR makeover for a 21st century audience that doesn’t know its practitioners, what they do or why they do it.

Ideologues are out there, their anti-science messages fueled by social media, bellowing nonsense and with a few bullying the scientific community. The immediate thought is to invoke scientific neutrality—science is apolitical, neutral, value-free, so leave us alone. Wrong wrong wrong!

Scientists are political, non-neutral, they have values, their work is driven by them. They enter and do science with a clear mindset about both how to do science and why. The response to ideological bullies and the belittling of scientific findings is not to plead purity and disengagement but just the opposite: engagement, advocacy, a clear sense of accuracy and validation of results, a love for methods that over time demonstrably produce reliable results, a sincere humility in the face of fallibility—these virtues generate trust. Scientists who are seen as humans, who are relatable, who are engaged members of their community, who can advance the values of science, who care deeply about their work and its meaning for others will be both heard and trusted. To rebuild trust, science must bare its soul.

## Supplementary information

Peer Review File
