# Supplementary material for: Soul Men and Women—what must science do to regain public trust?
Source: EMBO Rep. 2024 Nov 20;26(1):1–2. doi: 10.1038/s44319-024-00325-0 (PMC11724047; doi:10.1038/s44319-024-00325-0)
Supplement: Supplementary file 1 — Peer Review File [file 44319_2024_325_MOESM1_ESM.pdf]

EMBO reports

Soul Men and Women - What must science do to regain public trust?

Arthur Caplan

Corresponding author: Arthur Caplan (Arthur.Caplan@nyumc.org)

Review Timeline:

Submission Date:	6th Nov 24
Accepted:	11th Nov 24

Editor: Holger Breithaupt

Transaction Report:

This article type is not peer reviewed.
